# Predictors of treatment duration in conservative management of developmental dysplasia of hip -a retrospective cohort study

**DOI:** 10.1007/s00402-024-05715-6

**Published:** 2024-12-27

**Authors:** Tanja Kraus, Anita Hammerschmid, Bernhard Guggenberger, Michael Novak, Gudrun Schappacher-Tilp, Martin Svehlik

**Affiliations:** 1 Medical University of Graz, Graz, Austria; 2 FH JOANNEUM - University of Applied Sciences, Graz, Austria

**Keywords:** Conservative management, Developmental dysplasia of the hip, Sonography, Treatment time

## Abstract

Developmental dysplasia of the hip is a prevalent condition in newborns. However, predicting the duration of conservative treatment remains challenging. This study aimed to determine the duration of treatment more precisely by analyzing associated factors. We conducted a retrospective analysis and developed a linear regression model based on 503 patients treated at our institution over the last 10 years. A linear regression model (GLM) was used for predicting treatment duration (df residuals 371, df model 3, Pearson Chi2 78.9, Number of iterations 15). The baseline scenario thereby feature a child with an average age at the beginning of treatment (35th day of life), both sides pathologically affected, and a minimum alpha angle of 29 degrees. The GLM identified age at treatment onset, alpha angle, and bilaterality as significant predictors of treatment duration. A four-week delay in treatment initiation extended the duration by one week, while a 5-degree increase in the alpha angle reduced it by two weeks. Bilaterality added 19 days to treatment duration. However, sex and clinical hip instability did not significantly affect the treatment time. These findings enable the calculation of treatment duration based on identified factors, potentially improving the management and planning of conservative therapies for developmental dysplasia of the hip in newborns.

## Introduction

Developmental Dysplasia of the Hip (DDH) is the most common congenital disorder in newborns, potentially leading to significant disability later in life. Its incidence varies widely, ranging from 1 per 1000 live births to as high as 76 per 1000, contingent upon the chosen definition, geographical region, and specific population under consideration [1]. Contributing factors include breech presentation, female sex, and a positive family history [2].

Untreated DDH can result in limping, joint stiffness, pain, and abnormal gait. It remains a significant cause of up to 30% of primary total hip replacements in individuals under 60 years old [3]. DDH involves an abnormal relationship between the femoral head and the acetabulum, ranging from mild acetabular dysplasia to frank dislocation. According to Mulpuri diagnosis and treatment during childhood vary based on severity, age at diagnosis, and professional opinion [4]. However, the primary treatment goal is to achieve and maintain a stable reduction of the femoral head in the acetabulum.

Early intervention is paramount to maximizing the remaining growth potential and preventing complications linked to delayed treatment and surgical intervention. Screening and surveillance programs for newborns, utilizing ultrasound, enable early detection of DDH, facilitating successful conservative treatment in nearly all cases. For example, Biedermann’s study of 28,092 neonates found a treatment rate of only 1%, with just 0.04% requiring open reduction; the majority was effectively managed conservatively [5].

The underlying cause of DDH is growth disturbance at the acetabular growth plate. To facilitate hip maturation in newborns, a crucial strategy involves rebalancing the acetabular growth plate by positioning the hip in flexion and abduction.

This involves maintaining flexion of over 90° combined with slight abduction of 30–60° (referred to as the “human position” by Salter or Fettweis [1, 6]), which ensures a perpendicular force to the growth plate, optimizing growth potential, and reducing the risk of avascular necrosis (AVN).

Managing neonatal DDH therefore involves flexion–abduction treatment, utilizing splinting or casting (depending on severity and hospital protocol. This treatment continues until the affected hip achieves concentric stability and maturity. Despite seeming straightforward, neonatal DDH management poses significant challenges. It’s often unseen by parents initially, with its severity and lifelong impacts not immediately apparent [7].

Typically painless with unrestricted hip movement, DDH can be managed conservatively, though the unpredictable treatment duration often challenges parental compliance. This study aimed to develop a tool for predicting the duration of successful neonatal DDH management, based on extensive data from treated newborns.

## Methods

We conducted a retrospective 10 -year study of patients screened and managed for DDH conservatively at our Paediatric Orthopaedic Unit.

In our country, hip ultrasound is integrated into the national surveillance program for newborns, ensuring regular screening for DDH. These ultrasound examinations are conducted within the first five days after birth and repeated between the 4th and 7th week of life to coincide with the developmental nature of DDH. The Graf-method is uniformly utilized in all ultrasound assessments, primarily administered by paediatricians. In cases where diagnostic uncertainty arises during the initial examinations, consultation with a paediatric orthopaedic surgeon is sought. Hips, showing any signs of pathology or requiring treatment based on the Graf classification are referred to the paediatric orthopaedic surgeon. Upon independent evaluation, treatment is initiated. The primary objective is to monitor the child until a concentric reduction of the femoral head in the acetabulum is achieved, with an alpha angle of at least 60 degrees (designated as type I or mature hip according to Graf).

Treatment decisions depend on the child’s age and the angles observed during ultrasound examinations, determining whether the hip requires treatment or observation.

Treatment decisions are based on the child’s age and ultrasound-determined angles, which determine whether the hip needs treatment or can be monitored.

### Graf`s treatment algorithm

Following Graf’s treatment algorithm [8], immature hips (Graf type IIa, IIa+) undergo ultrasound surveillance until complete maturation (Graf type I) is achieved, without requiring treatment. Dysplastic hips (Graf type IIc hips younger than 3 weeks of age, IIa – and IIb hips) receive treatment with a splint, worn in a 110° flexion and 45 to 60 degrees abduction position. The splint is worn continuously until complete maturation (Graf type I) is attained, with regular ultrasound checks every 2 to 6 weeks.

Dislocated hips (types IIc unstable in children aged over 3 weeks, type D, III, and IV) had to undergo reposition and retention until a concentric hip with an alpha angle of at least 60 degrees was reached [8].

### Devices used at the paediatric orthopaedic unit

For flexion abduction treatment in stable immature hips, the Tübingen^®^ splint (Otto Bock HealthCare Deutschland GmbH) was used. It has to be worn 24 h/7days per week until the hips reach full maturation (type I according to Graf), with regular ultrasound checks in two to four-week intervals.

Dysplastic hips which required reposition and retention, were treated with a spica cast for four weeks after an initial overhead extension of 5–10 days. General anaesthesia was administered for hip reduction after arthrography, and the cast was applied in a position with 110 degrees of flexion and 45 to 60 degrees of abduction. After four weeks, the cast was removed, and a Tübingen splint was applied, worn 24 h/7days per week until the hips reached full maturation (type I according to Graf), with regular ultrasound checks in two to four-week intervals. This approach was chosen until 2017. From 2018 cast treatment for instable dysplastic hips was replaced by Pavlik harness treatment and only in the rare case of failures a cast is used. The Pavlik harness is used until Graf type I hips are achieved. Treatment is switched to a cast when there is no improvement of alpha angles within three months after treatment start.

### Data collection

Patients who had a hip ultrasound examination and who were treated conservatively for DDH in our paediatric and adolescent orthopaedic unit between 2007 and 2017 were included in this study. Data was collected retrospectively using a computerized documentation system based on the SAP healthcare solution [9].For each patient demographic data (sex, age), the number of affected hips (unilateral, bilateral), the sonographic measured hip angles and the result of the Ortolani test for hip in/stability was collected. For premature birth chronological age was corrected according to the gestational age. All included individuals were required to have a minimum of two recorded hip datasets at our unit at different time points.

### Inclusion criteria

The study included otherwise healthy children with one or both hips showing immaturity or dysplasia. Exclusion criteria included patients with underlying conditions (e.g., syndromes, neurological disorders, endocrine diseases) and those with mature hips (Graf type I) on both sides at the initial visit. Additionally, patients with incomplete data were excluded.

### Statistics

### The generalized linear regression model

We developed a predictive model to assess the duration of treatment, examining factors such as age, sex, alpha angle, the number of affected sides, and clinical instability tested by the Ortolani–Barlow manoeuvre. For statistical reasons and to achieve a higher group size for calculations, the hips were grouped based on immaturity and severity of DDH (see Table 1).

**Table 1:** Hips were diagnosed according to the Graf method. The different hip types were pooled in three groups of which the pooled group “NPH” represents non pathological hips. This group includes physiological immature hips as well as mature type I hips resulting from patients with only one side pathology. The “DHC” group includes dysplastic but centered hips while the “DHD” group includes hips at risk (critical) as well as decentered hips. *sonographically

Type according to Graf	In accordance with	Pooled as group
I, IIa, IIa+	Non – Pathological Hips	NPH
IIa-, IIb, IIc-stable	Dysplastic Hips, Centered	DHC
IIc-instable*, D, III und IV	Dysplastic Hips, Decentred	DHD

The predictive model was based on a generalized linear regression model (GLM) assuming a gamma distribution for the response variable and the identity link function. The significance of each coefficient in the model was confirmed using the Wald- test. This test validated the factors of age, sex, alpha angle, number of affected sides, and clinical instability as essential predictors.

We established a baseline scenario, featuring a child with an average age at the beginning of treatment (the 35th day of life based on our data), both sides pathologically affected, and a minimum alpha angle of 29 degrees. By modifying factors such as the degree of pathology or the age of the child, the model provides customized predictions for treatment duration.

Although we considered other potential factors like sex and clinically tested hip instability for their possible contributions, their inclusion did not enhance the predictive power of the model. This suggests that the number of affected hips, the age at the start of treatment, and the alpha angle are the key determinants of treatment duration.

In addition to our primary analyses, we conducted an exploratory data analysis to better understand the underlying data structure. The results of this analysis are presented in various formats to provide a comprehensive view of our findings. Frequency tables were used to display the distribution of categorical variables, while continuous variables were described using measures of central tendency and dispersion. Depending on the nature and distribution of the data, we presented these variables as mean with standard deviation or as mean with range, selecting the most suitable format for each specific context.

## Results

**Fig. 1:**
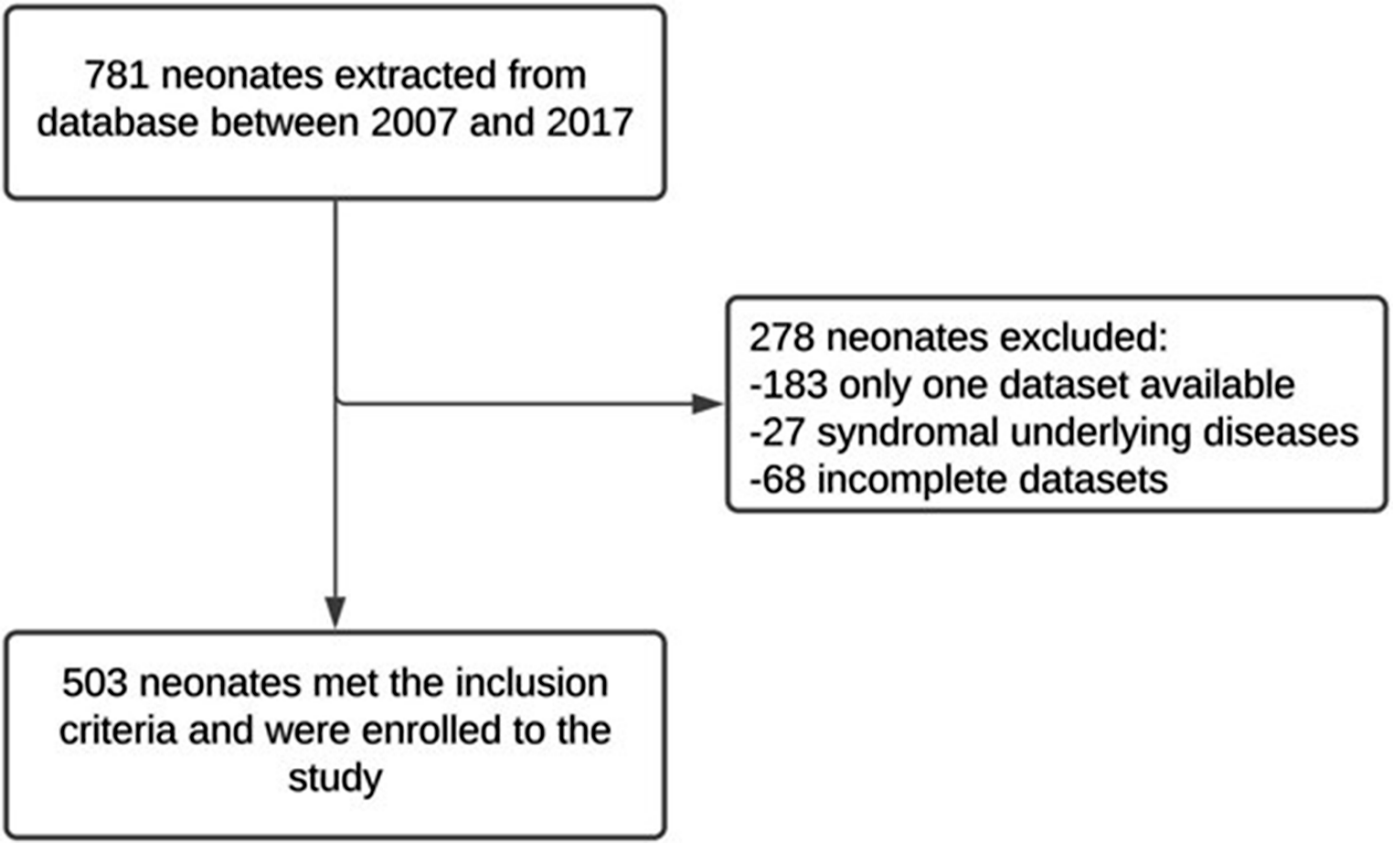
The figure depicts the stepwise selection process for the retrospective study, including total records screened, exclusions based on predefined criteria, and the final cohort of patients meeting inclusion requirements

Within our 10 years report 781 neonates had hip ultrasound examinations at our unit. 503 patients, 86 males and 417 females, with one or both hips affected, were included in the study while 278 were excluded. In 183 of the excluded infants only one data set was available, 27 showed syndromal underlying diseases (Achondroplasia (*n* = 4), Trisomy (*n* = 9, Ehlers Danlos (*n* = 2), Osteogenesis imperfecta *n* = 3, others (*n* = 9)) and in 68 infants’ data sets were not complete (Fig. 1).

The average age at presentation was 35 days, ranging from 0 to 265 days (median = 27, std = 34.75). Left hip involvement was more frequent than right hip involvement, as detailed in Table 2.

**Table 2:** Distribution of the different hip types in absolute numbers and in percentage according to Graf and the assigned pool group for statistical calculation (NPH = non pathological hips; DHC = dysplastic hips, centered; DHD = dysplastic hips, decentered

Hip Type according to Graf	Right side (%)	Left side (%)	Total number of hips (%)	Assigned group
I	120 (23.9)	79 (15.7)	199 (20)	NPH
IIa	156 (31)	157 (31.2)	313 (31)	NPH
IIa+	47 (9.3)	57 (11.3)	104 (10)	NPH
IIa-	36 (7.2)	48 (9.5)	84 (8)	DHC
IIb	13 (2.6)	23 (4.6)	36 (4)	DHC
IIc instable	7 (0.4)	11 (2.2)	18 (2)	DHC
IIc stable	41 (8.2)	49 (9.7)	90 (9)	DHD
III	55 (11)	56 (11)	111 (11)	DHD
IV	1 (0.2)	5 (1)	6 (1)	DHD
D	27 (5.4)	18 (3.6)	45 (4)	DHD
**Total**	**503** **(100%)**	**503** **(100%)**	**1006** **(100%)**	

Among the DDH hips, 42 exhibited clinical instability, while others did not, despite decentration. Interestingly, 12 hips in the immature group also displayed a positive Ortolani-Barlow sign, even with age-related ultrasound examinations appearing unsuspicious.

Out of the 503 patients, 128 constituted a monitoring-only group without receiving treatment, while the remaining 375 underwent treatment. The average treatment duration was 10 weeks, with a range from 4 to 32 weeks.

While exploring other potential factors like sex and clinically tested hip instability, their inclusion did not enhance the model’s predictive power. This suggests that the number of affected hips, the age at the start of treatment, and the alpha-angle are the key determinants of treatment duration. Additionally, an exploratory data analysis was conducted to better understand the underlying data structure.

A noteworthy finding was the significant impact of the patient’s age at the start of therapy on treatment duration. Younger children tended to have shorter treatment durations. The GLM further supported this relationship, indicating that a four-week delay in initiating therapy prolonged treatment time by almost one week (see Table 3). Conversely, starting treatment earlier corresponded to a reduction in treatment duration.

**Table 3:** Results from generalized Linear Model (GLM) regression for predicting treatment duration (df residuals 371, df model 3, Pearson Chi2 78.9, number of iterations 15). The baseline scenario features a child with an average age at the beginning of treatment (35th day of life), both sides pathologically affected, and a minimum alpha angle of 29 degrees. All predictors were statistically significant (p-values < 0.05) as determined by the Wald test

Parameter	Coefficient	Standard error	Wald-Test		
			95% confidence interval		
			Lower limit	Upper limit	*p*-value
Constant	164.5727	9.3969	146.209	182.936	< 0.001
One side pathological	-18.8505	6.087	-30.780	-6.920	0.002
Centered age (days)	0.1441	0.064	0.019	0.27	0.024
Alpha-centered	-2.9357	0.411	-3.741	-2.130	< 0.001

The GLM also revealed that the alpha-angle is a significant predictor for treatment duration (see Table 3). An inverse relationship between the alpha-angle and treatment duration was observed, where lower alpha-angles corresponded to longer treatment durations. With the lowest alpha-angle in our patient population at 29 degrees, the model predicted that for every 5-degree increase in the alpha-angle, the treatment duration would decrease by about 2 weeks.

The number of pathologically affected sides emerged as another significant factor associated with treatment duration. Specifically, in cases of unilateral pathology or one-hip involvement, the model predicted a decrease in treatment duration by approximately 18.85 days. The independence of these factors allows the evaluation of each factor’s effects in isolation, without the need to account for potential confounding or interaction effects between them. The cumulative effect on treatment duration can be computed by summing the individual effects of these factors.

$$\eqalign{ duratio{n_{o{f_{treatment}}\left( {days} \right)}}\, & = \>164.5727 + \>0.1441*\left( {age\> - \>mea{n_{age}}} \right) \cr & - 2.9357*\left( {alph{a_{angle}} - \>mi{n_{alph{a_{angle}}}}} \right) \cr & - 18.8505*Patho \cr} $$

where $\:Patho=0$ if both sides are pathological affected and 1 else. Age is the patient’s age at start of the treatment.

## Discussion

Our study examined the influence of factors associated with DDH on the treatment time.

The factors considered were age, sex, alpha angle, the number of affected hips (unilateral or bilateral), and hip stability proofed by the Ortolani test. Age and sex were chosen as known and already published factors [2, 4, 10, 11]. Moreover, according to our clinical experience, we expected the alpha angel and bilaterality as an important factor associated with the length of the treatment. Based on our large data set, significant influences on treatment time were observed for age, alpha angle, and bilaterality, while sex and clinical hip instability showed no discernible impact. A linear regression model was devised to predict treatment duration based on these factors. The derived formula allows prediction of treatment duration in newborns with developmental dysplasia of the hip.

Untreated DDH can lead to abnormal gait, pain, and early osteoarthritis, remaining the primary cause of total hip replacement in individuals under the age of 60 [3].

The introduction of hip ultrasound screening in newborns has significantly decreased the need for surgical hip interventions during growth [12–14]. Moreover, early detection during the newborn period has contributed to an exceptionally high success rate in treatment outcomes [14, 15]. While conservative treatment for DDH in early life is typically shorter than surgical interventions with postoperative rehabilitation in later stages, the treatment duration can vary between individuals and remains unpredictable.

The mean treatment duration in our patient cohort was 10 weeks. Comparable treatment durations were reported by Bialik (11.5 ± 7.9 weeks, ranging from 1 to 45 weeks) and Salduz (11.7 ± 6.2 weeks) [16, 17]. Additionally, Atalar et al. reported a mean treatment duration of 8 weeks (with a range of 5–11 weeks) in their study [18].

In contrast, Kokavec et al. observed a mean duration of 6.1 months in their conservatively treated patients, with a success rate of 65% in dysplastic hips and 34% in dislocated hips [19], while Kitoh et al. reported a mean duration of their conservative treatment of 11.2 ± 22.3 months [20]. It’s noteworthy that in both studies, the age at the onset of treatment was relatively higher, ranging from 3 to 8 months in the study by Kokavec and 3.9 ± 1.08 months in the study by Kitoh.

The differences in starting ages for measuring alpha angle improvement across various studies inherently affect the time required to reach an alpha angle of 60 degrees. This variation is due to the differing growth rates at different ages [21]. Therefore, comparisons between studies with differing starting ages for measuring alpha angle improvement should be made cautiously. The lack of comparability is also mirrored by the fact that current approaches for detection of DDH vary globally, lacking uniform agreement, as highlighted in the literature [22].

Finally, the rapid hip maturation observed in newborns within the initial 12 weeks aligns with natural growth patterns, emphasizing the need for controlled studies to isolate treatment effects accurately.

Age significantly influenced treatment duration in our study. Early hip screening enabled prompt DDH detection despite a mean age of 35 days at treatment onset.

Delaying treatment was associated with longer treatment durations. However, this finding should be interpreted with caution, as the rapid hip maturation observed within the initial 12 weeks, as noted by Tschauner et al. (1994), may be influenced by natural growth patterns rather than the treatment itself [10].

Cheng et al. (1994) observed a similar maturation trend in stable Graf hips aged ≤ six months [23]. Additionally, Wilkinson et al. (2018) noted a notable increase in the α angle during the first four months of life [24]. These findings suggest the crucial hip plasticity window within the initial three months of life. Properly treated DDH during this period can likely revert to normal development within is inherent plasticity [25]. Early diagnosis is pivotal, emphasizing a key message for parents and caregivers [26]. According to our linear regression model, a one-week delay in diagnosis necessitates four additional weeks of therapy.

Extended conservative treatment durations often coincide with decreased parental adherence to prescribed medical devices, particularly concerning DDH [27]. DDH’s asymptomatic nature and unrestricted hip movement make it inconspicuous for parents. As the child ages, parents may hesitate to use orthoses, fearing they may impede motor development. Consequently, parental compliance with orthoses decreases with the child’s age, impacting treatment success [27]. Predicting conservative treatment duration could bolster compliance, alleviate parental stress, and ultimately enhance treatment success. With this retrospective study, we investigated factors, associated with treatment time in conservative DDH management. We developed a formula which might be used by the clinician to inform the parents about the length of treatment in conservative DDH management. While alpha angle and bilateral pathology are intrinsic factors, the age at diagnosis is modifiable and may influence treatment duration. Therefore, we propose the early detection of DDH as beneficial in potentially reducing the duration of conservative therapy. Additionally, improved management of outpatient’s controls could reduce the socio-economic costs of the treatment.

Our study aimed to identify factors influencing treatment duration, providing data for calculating individualized treatment times. This offers clinicians a valuable tool for accurately predicting the duration of DDH treatment.

Our data revealed that treatment duration is associated with the alpha angle significantly influences treatment duration in infants with DDH, representing the bony acetabulum’s condition. Lower alpha angles correlate with compromised acetabular development, leading to prolonged treatment.

For every five-degree increase in the alpha angle above the 29-degree baseline, treatment duration decreases by 5 days, consistent with findings by Salduz et al. (2018) [17]. However, while the correlation between the alpha angle at the start of treatment and growth to an angle of 60 degrees aligns with expected natural growth patterns in younger patients, it is important to note that this relationship should be interpreted with caution. Due to the absence of a control group, we cannot definitively attribute this growth to the treatment itself.

Bilateral pathology increases treatment duration by approximately 2 weeks, although literature on the duration of early bilateral DDH treatment is limited. Studies report varied outcomes with Pavlik Harness therapy for bilateral cases, but there is no clear explanation for the extended treatment times. Abnormal movement patterns associated with hip dislocation may play a contributing role. Our study found no gender-based difference in treatment duration, contrary to previous reports associating lower alpha angles in females with prolonged treatment. The absence of gender difference aligns with Salduz’s findings and may stem from our population’s young age at diagnosis (mean 8.8 weeks).

A low alpha angle, bilateral pathology, and older age at diagnosis are associated with longer durations of conservative DDH treatment. While alpha angle and bilateral pathology are intrinsic factors, the age at diagnosis is modifiable and may influence treatment duration. Therefore, early detection of DDH might be beneficial in potentially reducing the duration of conservative therapy. However, to confirm this effect, a randomized controlled trial would be necessary.

It’s important to acknowledge the limitations of our study. Firstly, it is a retrospective analysis conducted at a single paediatric orthopaedic centre, which might affect the generalizability of the findings to a broader population. Moreover, we did not considerate on growth data in this study. Additionally, the calculation of treatment time is based on the available data, and the number of included hips is moderate, potentially impacting the robustness of the results. Another further limitation is the absence of external validation, which is crucial for evaluating the generalizability of our findings. It will be a focal point in future studies. Therefore, the observed values and associations should be confirmed through prospective studies and broader clinical practices to establish the reliability and applicability of the calculated values.

Despite these limitations, our study provides valuable insights into factors associated with the treatment duration of DDH, offering a foundation for future research and clinical considerations.

## Conclusion

In conclusion, our study highlights that the age at the initiation of treatment, the alpha angle, and bilateral hip involvement are key factors significantly associated with the duration of treatment for DDH. Conversely, factors such as the patient’s sex and the presence of clinical hip instability did not exhibit a significant impact on the treatment duration. These findings contribute valuable insights into the determinants of treatment duration for DDH and provide a basis for informed clinical decisions and interventions.

## Data Availability

Data can be obtained by the authors on reasonable request.
